# Supplementary material for: CAST as a Potential Oncogene, Identified by Machine Search, in Gastric Cancer Infiltrated with Macrophages and Associated with Lgr5
Source: Biomolecules. 2022 May 6;12(5):670. doi: 10.3390/biom12050670 (PMC9138541; doi:10.3390/biom12050670)
Supplement: Supplementary file 1 [file biomolecules-12-00670-s001.zip › biomolecules-1370921-supplementary.pdf]

Supplementary Table S1

(The ratios represent high/medium expression)

	HPA036881	HPA036882	CAB009491
**Breast cancer**	7/10	6/11	10/12
**Colorectal cancer**	6/11	4/11	9/11
**Urothelial cancer**	6/11	7/11	7/11
**Cervical cancer**	6/11	9/11	5/11
**Thyroid cancer**	2/4	2/4	4/4
**Stomach cancer**	6/12	2/12	9/12
**Renal cancer**	6/12	4/11	9/12
**Prostate cancer**	6/12	10/12	12/12
**Liver cancer**	5/11	1/11	5/10
**Pancreatic cancer**	4/11	3/10	9/12
**Melanoma**	4/11	2/11	9/12
**Skin cancer**	3/11	8/12	11/12
**Lung cancer**	3/12	6/12	6/10
**Head and neck cancer**	1/4	2/4	2/4
**Ovarian cancer**	2/12	3/11	8/12
**Testis cancer**	1/10	0/10	1/11
**Endometrial cancer**	1/11	1/11	3/11
**Glioma**	0/10	0/10	2/12
**Carcinoid**	0/3	1/3	3/4
**Lymphoma**	0/12	2/12	0/12
